# Grand challenges in cellular biochemistry: the “next-gen” biochemistry

**DOI:** 10.3389/fchem.2014.00022

**Published:** 2014-04-29

**Authors:** Cecilia Giulivi

**Affiliations:** ^1^Department of Molecular Biosciences, University of California, DavisDavis, CA, USA; ^2^Medical Investigations of Neurodevelopmental Disorders Institute, University of California, DavisDavis, CA, USA

**Keywords:** biochemistry, history, RNA, enzymes, instrumentation, advances, discoveries, one-health

It is said that Biochemistry is a young scientific discipline, making its “formal” debut toward the end of the 1900th century (Manchester, 2000), with seminal works by Buchner (1897, Jaenicke, 2007), (Pasteur and Berthelot, 1906), Hill (1898), Embden and Glaessner (1901), Meyerhof (1911), Parnas (1911), Harden (1911), and, of course, Michaelis and Menten (Johnson, 2013; Michaelis and Menten, 2013; Deichmann et al., 2014). These early and important contributions marked the road for future work in the fields of (a) chemical and biochemical structures and associated functions by Sanger (1945), Perutz (1942), Franklin (1950), Watson and Crick (1953), Pauling et al. (1949, 1951), Pauling and Corey (1951), Zuckerkandl and Pauling (1962), Kornberg (1974, 1977), Boyer (1997), Walker et al. (1982), Abrahams et al. (1994); (b) metabolic pathways and regulation by Ochoa and Valdecasas (1929), Krebs and Johnson (1937), Novelli and Lipmann (1947), Fischer et al. (1959), Cori and Cori (1923, 1925), Houssay (1945, 1948), Lehninger (1942, 1945), Caputto et al. (1949), Cardini et al. (1950), Mitchell (1961), Benson and Calvin (1947), Hershko et al. (1980), Hershko and Ciechanover (1992), and (c) contributing to innovative techniques or approaches dedicated to advance basic knowledge (and making our lives easier) with Smith (1982), Winter et al. (1982), Mullis et al. (1986) (ante and post-PCR era) and Shimomura (1979), Chalfie et al. (1994), Heim et al. (1994) (ante and post-green fluorescent protein), Yalow et al. (1964), and Williams et al. (1977), Springer et al. (1979); (d) signaling molecules and signal transduction by Levi-Montalcini and Amprino (1947), Cohen et al. (1954), Sibley et al. (1986), Benovic et al. (1987), Frielle et al. (1987), Fargin et al. (1988).

Back in 1896, Buchner's preparation of a “juice” from yeast (Buchner, 1897) is often regarded as the birth of modern biochemistry. However, I tend to digress with this strict view of biochemistry, reasoning that we (as a species) were taking advantage of biochemical principles without having a deep understanding of the underlying molecular processes. For instance, consider Buchner's “juice” or actually wine making. This method, that has at its core the fermentation process one of the key pathways in biochemistry, dating back to around 6000 BC (Chambers and Pretorius, 2010). Refer to the complicated production of fish sauces considered among the most common flavor-enhancing condiments produced and distributed across ancient Roman Empire (Lowe, 2009). Another example comes from the mixture of organic preservatives (i.e., biochemical) used for ancient Egyptian mummification (Buckley and Evershed, 2001). Or think about the effects of diet on health as recognized by Hippocrates (460–377 BC; Caramia, 2006), the arab physician Ibn al-Nafis (Al-Nafis, 13th century) and Leonardo da Vinci (1452–1519; Caramia, 2006) as well as the experimentation with animals and structure—function of human body set by the Medieval Islamic era as early as the 9th century (Abdel-Halim, 2011). This early biochemistry was empirical, done in settings other than laboratories, serving immediate needs, and some passed onto next generations by oral traditions. Then we would reason, are these contributions valuable to the genesis of biochemistry? Should they be dismissed because the microorganisms were not genotyped, the reactions were done in dolia instead of microplates? Then, if we accept these very early facts (and why not experiments?) as part of the genesis of this field, we will need to accept that biochemistry is a long, long (ancient?) journey that has accompanied us since the dawn of civilization.

The general field of Biochemistry has grown since then to the point that it has been expanded to various more specific areas of research. For example, Cellular Biochemistry is at the crossroads of Chemistry (Organic, Physical, Analytical, Inorganic, Biological) and Biology (Chemical, Molecular) including studies on biomolecular structures and the mechanism of biochemical reactions, but also on the biological purposes of biochemical phenomena, i.e., metabolic pathways and their control, physiological significance and clinical relevance of topics presented. The regulation includes protein and gene expression analyses as well as protein post-translational modifications, epigenetic controls, metabolite-control systems, and gene-environment interactions as well as cell-cell interactions. This field covers areas from fundamental biochemical principles (e.g., enzymology, macromolecule structures) in cell-free systems to pathways, their regulation, and integration in physiology, and how their disturbance could lead to a number of diseases.

While tremendous progress has been achieved, here are some of the aspects that we think needs additional attention in the next upcoming years.

## On proteins: hiding behind a post-translational modification

How many new post-translational modifications have been found after the relevant discoveries on protein phosphorylation and their impact on signal transduction pathways (Fischer, 2013)? Among them, ubiquitination (Schmidt and Finley, 2014; Schreiber and Peter, 2014), methylation (Afjehi-Sadat and Garcia, 2013; Clarke, 2013), sumoylation (Yang and Chiang, 2013), acylation (Storck et al., 2013; Running, 2014), glycation (Nedic et al., 2013; Sousa Silva et al., 2013), nitrosylation (Gould et al., 2013; Michelet et al., 2013), glutathionylation (Allen and Mieyal, 2012; Zaffagnini et al., 2012), tyrosine *O*-sulfation (Kim et al., 2005), dityrosine crosslinks (Giulivi et al., 2003), ADP-ribosylation (Dani et al., 2013), and lysine acetylation (Bernal et al., 2014; Cain et al., 2014; Dos Santos-Pinto et al., 2014; Wang et al., 2014). It is clear that in some of these examples the modifications accompanied a biochemical or metabolic process (e.g., fasting Yang et al., 2011). But, so far limited cases have demonstrated that a single protein modification on a given target modulated a protein activity, and as a result, a pathway (e.g., Nie et al., 2009), modified the protein subcellular location (e.g., Moeller and Fenton, 2012), or the fate (e.g., Spasser and Brik, 2012). Then, how many of these modifications normally found in a cell are truly relevant to a specific biological process (Catherman et al., 2014; Dos Santos-Pinto et al., 2014; Vaudel et al., 2014)? Are they silent by-standers, surrogate markers (Pimentel et al., 2012; Perluigi et al., 2014)? Is there a hierarchical order or a cross-talk among several protein modifications within a single protein? What are the purposes of these modifications (Moore and Gozani, 2014)? What if the modified protein is not a rate-limiting step of a given pathway and/or has a high turnover? In what cellular compartments or organs are these modifications found? To gain a better understanding of protein modifications, which enhance and extend the diversity of proteins beyond that encoded by DNA and the transcriptome, we need to clarify these questions to truly understand the meaning of regulation of pathways in biochemistry and their relevance in disease.

## On RNA and its landscape: the more the merrier

In the midst of enzyme isoforms, metabolite controls, post-translational modifications, compartmentalization, gene transcription, protein-protein interactions, microRNAs (miRNAs), or small non-coding RNAs have emerged as new post-transcriptional regulators of gene expression (Feng et al., 2014). To date, a myriad of diverse cellular events [cognition, synapsis, cell fate, plasticity, cancer (Asrih and Steffens, 2013; Clifford et al., 2013; Di Leva and Croce, 2013; O'Carroll and Schaefer, 2013; Feng et al., 2014)] have been claimed to be regulated by miRNA. As indicated above for novel protein post-translational modifications, we are just starting to unveil the cause-effect for a limited number of miRNAs. Thus, more research is needed to address the links between miRNA expression and miRNA-targeted genes, the association between miRNAs and messenger RNAs, how this new regulation works in association (or not) with others already present in the cell, and what role miRNAs might have played in phenotypic evolution (Akbari Moqadam et al., 2013; Luo et al., 2013; Marco et al., 2013). To complicate the story further, next generation sequencing technologies targeting the miRNA transcriptome revealed the occurrence of RNA fragments different from miRNAs. A growing evidence suggests that RNA fragments derived from small nucleolar RNA (snoRNA) and transfer RNA (tRNA) are neither RNA turnover artifacts nor random degradation products but rather stable species, which may have functional activity in the normal as well as in cancer cells (Falaleeva and Stamm, 2013; Lui and Lowe, 2013; Martens-Uzunova et al., 2013). But the story does not end here. Long non-coding RNAs (lncRNA) have been described to act as decoys of RNA-binding proteins or microRNAs and can compete for microRNA-mediated inhibition leading to increased expression of the mRNA (Louro et al., 2009; Whitehead et al., 2009; Yoon et al., 2013; Diederichs, 2014; Fatica and Bozzoni, 2014; Johnsson et al., 2014; Nakagawa and Kageyama, 2014). Future studies would need to decipher how these different messages (including circular RNA) are read by the cell, their role in regulating pathways or cellular processes, and their link to other regulatory systems (Tay et al., 2014).

## On inter-cellular communication: cell-derived membrane vesicles as the new molecular mercury

The relatively recent discoveries on the occurrence of cell-derived membrane vesicles (CVMs) add another layer of complexity to the field of cell-cell communication. CMVs have a biological cargo constituted by proteins, RNA or DNA, with the potential to change the phenotype of the receiving cell (Quesenberry and Aliotta, 2010; Rak, 2010; Lee et al., 2011; Sadallah et al., 2011). These vesicles are classified into exosomes, ectosomes, microvesicles, microparticles, apoptotic bodies, and are originated from different subcellular compartments. The molecular mechanisms regulating their formation, release and degradation are not fully understood; however, several studies highlight their role in tumor growth, microRNA delivery, atherosclerosis, pre-eclampsia, as well as their potential use as drug delivery (Aatonen et al., 2012; Biancone et al., 2012; Lee et al., 2012; Redman et al., 2012; Soleti and Martinez, 2012; van Dommelen et al., 2012; Vickers and Remaley, 2012; Camussi et al., 2013; Choi et al., 2013; Gonda et al., 2013; Inal et al., 2013; Martins et al., 2013; Principe et al., 2013; Loyer et al., 2014).

## On interdisciplinary approaches: are we listening— more than talking—to each other?

Theories have a transient nature; they last only until replaced by ones more consistent with the accumulated facts. To acquire “new information,” a great variety of methods must be used and their optimization or discovery of new methods will be an important area of research in the coming years. Studying isolated organelles, intact cells, tissues or organs may be seen as unphysiological and thus, requiring a considerable extrapolation of these results to animals including humans. Although these interpretations must always be made with caution, studies in intact organisms have the advantage of observing processes as a whole with the clear disadvantage that few of these processes occurring within it are accessible to study. In other words, while a considerable fractionation of the scientific problem may lead to the identification of key components, the way in which they interact may not be as in the intact organism. In this regard, data obtained *in vitro* and/or with simpler models have been used in dynamic mathematical modeling to promote a comprehensive understanding of *in vivo* complex mechanisms (Tummler et al., 2014). In addition to the advance of more accurate mathematical models, effort needs to be devoted at the development of *in vivo*, non-invasive techniques and methods to bridge the gaps between *in vitro* and *in vivo* systems (Zheng et al., 2011; Nandakumar et al., 2012; Ramkumar et al., 2013). The use of novel, non-invasive imaging techniques to follow, for instance, parasitemia (Maclean et al., 2013) or muscle oxygenation (Hamaoka et al., 2011) as well as fine-regulated molecular approaches such as optogenetics (Doll and Broadie, 2014; Sidor and McClung, 2014) or even more sensitive mass spectrometry technologies will be leading the future of Cellular Biochemistry. It also means to develop models that accurately mimic complex chemical systems as pioneered by Karplus (1959, 1963), Warshel and Levitt (1976), Chothia et al. (1989) and Levitt and Warshel (1975). Evidently, interdisciplinary approaches (which means interacting with colleagues in other fields) are needed to develop more sensitive and specific probes (chemistry), instrumentation (analytical instrumentation, computer engineering, bioinformatics), validation of targets (biochemistry) and delivery (nanomaterials, pharmacology, medicinal chemistry) to be applicable to medicine.

The interdisciplinary approach has a strong influence on the transition into “personalized medicine” which essentially requires the integration of various molecular approaches (proteomics, metabolomics, genome sequencing, imaging, to name a few) with the idea of providing the most effective therapy, minimizing side effects, and shortening treatment periods (Nandy et al., 2014; Stenson et al., 2014). One of the next challenges will be to integrate these interdisciplinary approaches in the clinics and making them affordable to all patients (Johnson et al., 2012).

## On the organism of choice: welcome to the battleground

Studies on simpler organisms such as worms, flies, yeast could be seen as somehow irrelevant to those in higher organisms, particularly with the wide use of murine models (Russell, 2013). However, if the question or problem resides within a highly conserved pathway across these species, the use of “relatively” simpler systems gives a unique opportunity to address a complex problem (Bednarova et al., 2013; Dassati et al., 2014). Studying these conserved or homologous processes across species and their modifications in front of environmental exposures will also integrate the field of gene-environment interactions (Napoli et al., 2013) and their impact on human disorders such as autism and obesity (Razquin et al., 2011; Schmidt et al., 2012; Galbete et al., 2013; Napoli et al., 2013; Lyall et al., 2014) as well as the evolutionary pressure to best suit the need (for example, Gnankine et al., 2013). In this context, an area that needs to be addressed is how we are re-defining our choices when selecting a biological study model to answer a particular question. For instance, the relevance of selecting a murine genetic background (even if inbred) that will not interfere with the issue of interest (e.g., Bourdi et al., 2011; Ulmasov et al., 2013), and still be reasonable breeders with relatively good health. Looking beyond the traditional murine models to uncover human-like diseases opens the door to collaborations between veterinarians and basic researchers (for example, Vernau et al., 2013) and explore new therapies (Patel et al., 2011; Nielsen et al., 2014), including the use of stem cells (Volk and Theoret, 2013) and “personalized medicine” (Palotie et al., 2013). Across-species studies fall under the umbrella named “One Health” initiative, which since 1984 combines human, animal, and environmental components to addressing global health challenges (Bidaisee and Macpherson, 2014). To this end, we will need to look up from our laboratory bench and try to understand the basis of diseases within ecosystems, reaching out to global health (Gutierrez et al., 2012; Conrad et al., 2013; Miller and Olea-Popelka, 2013).

## Concluding remarks

Progress in science is made either from discovering new facts or from re-interpreting well-established ones. In line with this concept, we should provide a balanced account of controversial areas and consider that the presentation of different views is important to reinforce the point that theories or hypotheses must always be open to reinterpretation with the advancement of knowledge [e.g., the use of vitamin D on osteoporosis (Reid et al., 2014)]. Indications of doubt raised by published studies provide impetus for further research by students, researchers, and clinicians. As processes evolve once they exist, it is our goal to shape the vision of Cellular Biochemistry adapting and evolving to the fast pace of scientific discoveries, trying to make this area the most suitable bridge between molecular biochemistry and medicine, placing it at the heart of translational medicine.

### Conflict of interest statement

The author declares that the research was conducted in the absence of any commercial or financial relationships that could be construed as a potential conflict of interest.
